# Managing Cognitive Decline Through a Social Robot–Based Intervention: Protocol for the engAGE Proof of Concept and Randomized Controlled Trial

**DOI:** 10.2196/67601

**Published:** 2025-09-02

**Authors:** Giulio Amabili, Elvira Maranesi, Arianna Margaritini, Anna Rita Bonfigli, Elisa Felici, Federico Barbarossa, Marco Benadduci, Laetitia Gosetto, Julie Guebey, Terje Grimstad, Riitta Hellman, Andrei Iulian Marin, Lars Thomas Boye, Ionut Manuel Anghel, Tudor Cioara, Roberta Bevilacqua

**Affiliations:** 1 Scientific Direction IRCCS INRCA Ancona Italy; 2 Division of Medical Information Sciences Hôpitaux Universitaires de Genève Geneva Switzerland; 3 KARDE AS Oslo Norway; 4 IRIS Robotics Iasi Romania; 5 TELLU AS Oslo Norway; 6 Technical University of Cluj-Napoca Cluj-Napoca Romania

**Keywords:** older people, older adults, mild cognitive impairment, social robotics, technology for elderly, digital health, cognitive training, innovation in health care

## Abstract

**Background:**

Dementia is challenging society in terms of the quality of life, the costs of health care systems, and caregivers’ burden. Dementia is often preceded by a status of mild cognitive impairment (MCI), during which a healthy lifestyle and cognitive therapy seem to be effective in counteracting the decline.

**Objective:**

The engAGE (Managing Cognitive Decline Through Theatre Therapy, Artificial Intelligence, and Social Robot–Driven Interventions) project aimed to build a technological platform to counteract cognitive decline in older adults with MCI through both cognitive therapy and lifestyle management.

**Methods:**

The engAGE platform was built around the social robot Pepper, which provides cognitive therapy. An activity tracker and a mobile app were also integrated within the platform to help older adults with MCI monitor their sleep and physical activity, in addition to offering cognitive games at home. The proof of concept (PoC) of engAGE was designed as a 6-month long randomized controlled trial (RCT) aimed to test the solution in three European countries: Italy, Switzerland, and Norway. During this period, under the supervision of a psychologist or a therapist, Pepper provided cognitive therapy on a weekly basis at health care or daycare facilities. In parallel, the use of the mobile app and the activity tracker was recommended on a daily basis. Participants were recruited through health care institutions and care organizations and evaluated through face-to-face interviews. The primary interest of the study was to assess the impact of engAGE on cognitive capacity through the Montreal Cognitive Assessment (MoCA) and the Memory Assessment Clinic Questionnaire (MAC-Q). In addition, changes in social engagement and quality of life were measured through the University of California, Los Angeles (UCLA) Loneliness Scale and the Warwick-Edinburgh Mental Well-Being Scale (WEMWBS). The PoC also focused on the acceptability and usability of engAGE, evaluated through the System Usability Scale (SUS) and the Unified Theory of Acceptance and Use of Technology (UTAUT) questionnaire. In addition, data were collected regarding the frequency of weekly sessions and access to the mobile app, as well as activity tracker measurements.

**Results:**

Data collection commenced in November 2023 and finished in July 2024, with the enrollment of 49 older adults with MCI (n=40, 81.6%, assigned to the experimental group [EG] and n=9, 18.4%, to the control group [CG]) conducted from October 2023 to April 2024. Data analysis was concluded in November 2024, and results will be published by 2026.

**Conclusions:**

The engAGE PoC represents an innovative study focused on the impact of a technology-based multidomain intervention designed for older adults with MCI that aimed to counteract cognitive decline through cognitive training, as well as improvement in terms of the quality of life, social engagement, physical activity, and sleep quality of primary end users.

**Trial Registration:**

ClinicalTrials.gov NCT06302686; https://clinicaltrials.gov/study/NCT06302686

**International Registered Report Identifier (IRRID):**

RR1-10.2196/67601

## Introduction

### Background

Dementia is a chronic neurodegenerative syndrome characterized by deficits in cognitive functions, associated with the loss of daily function and with mental and behavioral disorders. There are over 1 million people with dementia in Italy, of which 54% have Alzheimer’s disease and about 16% have vascular dementia [1]. The Swiss Federal Office of Public Health expects an increase in the number of people diagnosed with dementia to over 190,000 individuals by 2030 and to almost 300,000 by 2060 [2]. The same trend is expected in Norway, where the number of people with dementia is estimated to more than double from 2020 to 2050 [3]. According to the literature, cognitive interventions [4], regular physical activity [5,6], and reminiscence therapy appear to be effective in the treatment of people with mild-to-moderate dementia. Cognitive stimulation programs, computerized individual cognitive training, and physical exercise appear to be able to induce a significant improvement in cognitive performance, quality of life, and well-being in people with mild-to-moderate dementia [7-9]. Several studies show that, especially in the early stages of the disease, stimulation and participation in various types of activities can help counterbalance the cognitive changes related to the pathology, thanks to cognitive plasticity [10,11]. Neurocognitive disorders, in particular dementia, represent a major challenge for society. Efforts to reduce the burden for caregivers, as well as for society at large, are imperative. Older adults generally report higher preferences for their home over other living arrangements, and from the societal point of view this can contribute to the burden of care, for example, for their families. Dementia caregivers are at high risk of care burden, anxiety, and stress, which exposes them to a higher rate of mortality compared to noncaregivers [12]. Thus, promoting aging in place for people with dementia should not constitute a strategy to shift the burden of care from formal care services to informal caregivers. Instead, efforts should focus on reducing caregiver stress. Part of the difficulties and stress related to caregiving might be prevented by new information and communication technologies (ICT) and by developing innovative support services for these people. The introduction of innovative and cost-effective interventions to reduce the burden of dementia on public finances and individual families should be envisaged. Several digital interventions have been developed to address the needs of individuals with mild cognitive impairment (MCI), targeting cognitive stimulation, social engagement, and caregiver support. These include cognitive training apps [13,14], telehealth platforms [15,16], and social robot–based interventions [17,18]. Despite the growing availability of digital solutions, a significant gap remains in providing efficient, integrated, personalized, and adaptive home-based care for individuals with MCI. In fact, existing solutions often focus on single domains, rely on single technologies, and lack the ability to seamlessly integrate various components to address the complex needs of older adults with MCI.

### The engAGE Project

In this scenario, the engAGE (Managing Cognitive Decline Through Theatre Therapy, Artificial Intelligence, and Social Robot**–**Driven Interventions) project, cofinanced by the Ambient and Assisted Living (AAL) program of the European Union [19], aimed to counteract and slow down cognitive decline progression, enhance the intrinsic capacity of the users, and support the well-being of older adults with MCI by providing an ecosystem of services based on an innovative system that integrates social robots, mobile apps, and wearable sensors. Moreover, the engAGE project combined home-based intervention (daily usage of a mobile app and a wearable fitness tracker) with group cognitive training at a health care facility (weekly social robot–driven sessions). Thus, engAGE targeted the following challenges and needs for older adults with MCI, informal caregivers (family members), and formal caregivers (health care professionals):

- The project was primarily focused on older adults with MCI, aiming to improve their quality of life and well-being, allowing them to preserve their identity and reduce stress, memory loss, or communication challenges. The social robot can be a great tool in engaging older adults in these kinds of activities. It is always available and can provide verbal clues or suggestions according to each older adult’s wishes, needs, and memories. Moreover, the social robot may coach older adults to perform daily activities with greater independence (ie, coaching stepwise, prompting to complete activities at home).
- The project provides support to caregivers as well. Since caring for people with MCI puts a significant burden on informal caregivers, having the support of a technological platform can reduce anxiety, worry, and stress. Caregivers can personalize the content of the interventions to the wishes and preferences of older adults.

### Goal of the Study

This paper presents the study protocol of the engAGE proof of concept (PoC). The study aimed to demonstrate the viability of integrating social robot–based cognitive therapy in a health care facility with a mobile app and an activity tracker for daily use at home for counteracting cognitive decline in older adults with MCI. To provide more robust results in this sense, the study was designed as a 6-month long randomized controlled trial (RCT) in a real scenario in three European countries: Italy, Norway, and Switzerland. The study primarily evaluated cognitive capacity changes in older adults with MCI, in addition to assessing the impact on the quality of life and social dimension of primary end users, as well as the acceptability and usability of the engAGE platform. Moreover, the reduction in the stress and care load of formal and informal caregivers was measured. All measurements were performed at the beginning and end of the study to analyze intra- and intergroup differences. The novelty of this study design is the combination of at-home and in-place interventions to maximize the impact of cognitive stimulation through technology, minimizing the caregivers’ burden and encouraging the social connectedness among end users with the care network. The paper provides an up-to-date methodology of an interventional study to those researchers, scientists, and stakeholders who are interested in the use of social robots and ICT in health care and digital health. Finally, the paper discusses in depth the ethical issues concerning the adoption of social robots, machine learning (ML) algorithms, and health data management, along with the long-debated question of the informed consent form signed by people with MCI.

## Methods

### The engAGE Platform

**Figure 1:**
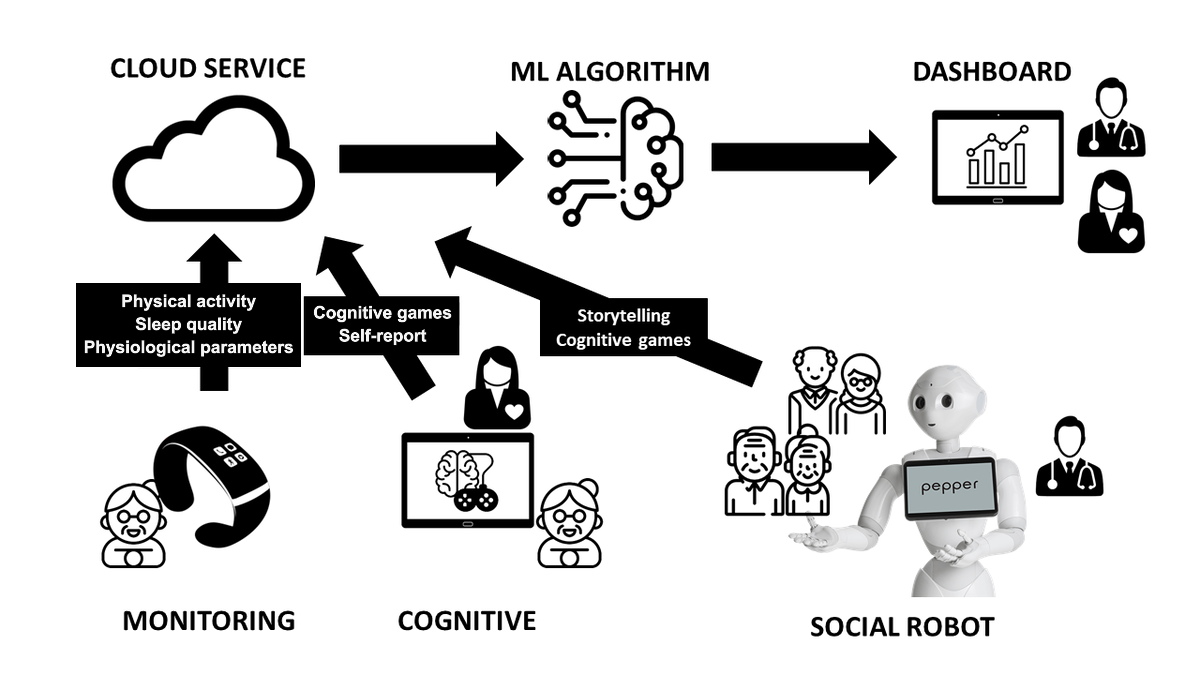
The engAGE platform. engAGE: Managing Cognitive Decline Through Theatre Therapy, Artificial Intelligence, and Social Robot–Driven Interventions; ML: machine learning.

The engAGE system offers 4 main services built around a social robot for self-managing and sustaining the cognitive function of older adults with MCI [20]: (1) holistic monitoring of daily life activities and perceived health state and well-being; (2) assessment of cognitive state and potential decline by leveraging ML algorithms; (3) social robot–based cognitive function support and coaching using cognitive games and storytelling scenarios; and (4) communication, cognitive stimulation, and personalization platform for end users. Figure 1 schematizes the engAGE platform services with the integration of social robots, sensor-based monitoring, and ML techniques. The primary end users (older adults) mainly interact with three devices: the social robot Pepper, a tablet, and a wearable fitness tracker (Fitbit). Formal caregivers supervise the execution of end users’ activities and help them, when needed. The interaction is through the device’s associated dashboards.

#### The Social Robot Service

As the social robot service, the Android version of Pepper’s tablet offers dashboards for cognitive games, storytelling, and activities to the end user through an easy-to-use interface. Equivalent cognitive games are also integrated into the communication service to allow the end user to play them on the tablet at home. Pepper is a humanoid social robot able to dialogue with the user by speaking, moving, and expressing emotions, being also equipped with an Android tablet placed at its chest. The user logs through the tablet at every session and then interacts with the robot and plays the planned activities (drama play or cognitive games). To track the user’s progress, each performance is recorded, and scores are sent to the cloud, along with the user ID. As the sessions are held in groups of 4 or 5 older adults, the users can choose to play individually or in teams. Once the type of game is selected, the user is redirected to a new screen in which all the games corresponding to the type selected is displayed. When the user plays and succeeds in a task, the robot congratulates them. To ensure that the user understands the robot, the words the robots says are displayed on the screen like subtitles. The interaction is recommended to be vocal as much as possible to improve the user experience. Different categories of cognitive games and storytelling activities are implemented in the service. The games are and will be iteratively developed and included in the service in the different versions of the prototype while considering end users’ feedback:

- Familiar games: crosswords, memory cards, and remembering objects that appear
- Quizzes: picture, musical, and cultural quizzes
- Story-/play-telling: story listening, poem recitation, and YouTube shorts, with questions asked about the activity

#### The Communication Service

The communication service is the hub of the entire system for providing dashboards for end-user interactions at home. It is a web app with several dashboards through which all the end users’ categories can communicate. This service is based on the MEMAS Life Mastering Assistant with the following functionalities that helps people with MCI: a calendar with reminders, step-by-step instructions for daily activities in the form of a series of images with spoken comments or videos, easy-to-access radio channels via streaming, photos with spoken comments and videos, music, easy-to-access network newspapers, cognitive games, weather forecasts, self-reporting questioners, and graphical results from the ML algorithms for secondary end users. The service is usable by both primary and secondary users. The primary user can be connected to several secondary users. In the administration module (a website page), the secondary user (ie, family member or formal caregiver) can manage an activity calendar, build albums with photos and videos, and configure access to favorite radio channels and newspapers. In general, the secondary user can edit the primary user’s interface to make using the service as simple as possible and tailored to the primary user’s needs. Data regarding self-assessment and cognitive games scores are sent to the ML service for further analysis. In this service also, the games are and will be iteratively developed and included in the service in different versions of the prototype while considering end users’ feedback: Sudoku, Mahjong, Minefield, crosswords, logics, math, Hangman, and memory cards.

#### The Monitoring Service

The monitoring service offers a tablet-based app where parameters such as the heart rate, sleep status, and physical activity (steps and distance walked) are monitored. These data are further used by the ML service as input for its algorithms. The monitoring service has little user interaction outside of the use of sensor devices. This service features a mobile app gateway that transfers data from the sensor device (Fitbit) into the cloud. In the first iteration, it transfers Fitbit data from the Fitbit service. It does so automatically while running in the background on the mobile device (Android tablet) of the primary user. Once the login and Fitbit authentication have been established, the app runs in the background, regularly polling data from the Fitbit service.

#### The ML Analytics Service

The ML analytics service has no direct end-user interaction; it gets data from the monitoring service and the communication platform and provides results to be displayed in the latter. The main goal of the service is to assess and correlate the daily physical activity, sleep parameters, game scores, and self-assessment data to offer a view of MCI progress [21]. It combines deep neural network algorithms/models to analyze objective activity data coming from the Fitbit wearable, with graph learning techniques that capture structural insights from subjective self-reporting activities to construct a better understanding of symptoms associated with cognitive decline.

### Study Design

The PoC was structured as a controlled longitudinal pilot study, with a before-and-after design, where observations were run on a series of enrolled individuals, who received the intervention described later, with data collected before and after the installation and use of the technical solution. The goal of trial evaluation was to demonstrate the viability of engAGE technology integration into everyday life to counteract cognitive decline. As secondary outcomes, the impact on the social dimension and quality of life of end users was evaluated. Moreover, acceptance over 6 months and the usability of proposed solutions were evaluated to understand the feasibility of the intervention. Despite the choice to structure the PoC as an RCT in order to provide more robust evidence on feasibility and efficacy [22,23], a formal sample size calculation was not performed. Practical constraints within the project were considered while deciding the number of end users to enroll. In fact, recruitment timelines, availability of rooms and professionals, and technical equipment represented practical limits for the study feasibility. After considering these constraints, the sample size for the PoC was set to 80 primary end users: 50 (62.5%) in the experimental group (EG) and 30 (37.5%) in the control group (CG).

In Italy, end users were recruited from the Neurology Unit and Alzheimer Assessment Unit (Memory Clinic) and from the Alzheimer daycare center of the Istituto di Ricovero e Cura a Carattere Scientifico – Istituto Nazionale di Riposo e Cura per Anziani (IRCCS INRCA). The research team could communicate with potential participants of previous projects and initiatives, as well as with other people who might have been be interested in contributing to the project evaluations. This method made it possible to be in contact with clients met before and with whom a relationship of trust was already established.

In Norway, participants were recruited from two daycare and housing centers in the city of Arendal. The first one, Solhaug, is a daycare center for old residents living in their own homes, while the second one, Plankemyra, is a combined housing and daycare center.

In Switzerland, end users were recruited with the help of a home assistance and care institution, the Geneva Institution for Homecare and Assistance (IMAD). The research team also reached the partner patient program for participants with potential MCI. All older adults interested in the study were considered for participation.

#### The Experimental Group

The EG used the engAGE system in two different settings: at health care organizations and at home. For the Italian site, the health care organizations were the Neurology Unit and Alzheimer Assessment Unit (Memory Clinic) and the Alzheimer daycare center of the IRCCS INRCA. Weekly sessions were run at the IRCCS INRCA usability lab (YOUSE) in the hospital. For the Norwegian site, weekly sessions were managed at Solhaug and Plankemyra in the city of Arendal. For the Swiss site, the participants (primary end users) attended weekly sessions at the Hôpitaux Universitaires de Genève’s (HUG) living lab. At the health care organizations, the primary end users interacted with Pepper. The interaction was supervised by tertiary end users (formal caregivers, ie, occupational therapists, psychologists, or psychotherapists) and included the following activities: dialoguing with the robot, storytelling, drama play, and cognitive and physical games. The human-robot interaction was planned to last about 1 hour and to be scheduled once a week for 6 months. At home, the older adults interacted with the tablet, supervised by informal caregivers (relatives). In addition, the primary end users played cognitive and physical games installed on the MEMAS app. This activity was suggested to be performed for 30 minutes every day for 6 months. Throughout the entire period of experimentation (unless they decided otherwise), the older adults wore the smartwatch for measuring their physiological parameters and steps. Any activity performed by them was assigned by formal caregivers and could be personalized, considering the primary end users’ abilities (difficulty levels of the games), lifestyle (reminding and monitoring services), and social interactions. Before starting the PoC, one session was dedicated to primary end users to teach them how to use the activity tracker and the MEMAS app at home. During weekly sessions, they were allowed to ask for clarifications about the use of these devices, and they could receive remote support from technicians during the week if any problem occurred.

**Figure 2:**
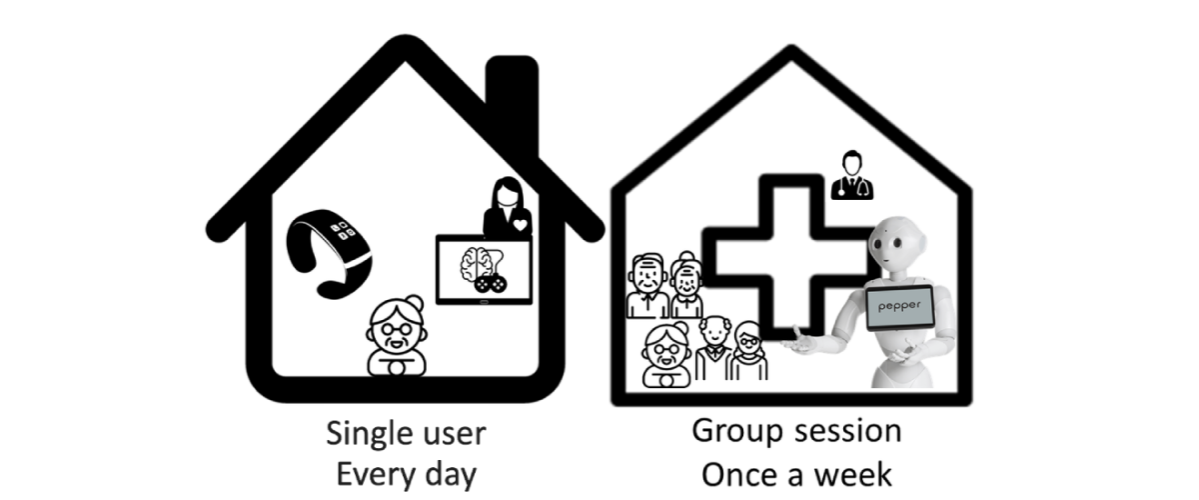
EG study design. EG: experimental group..

Figure 2 shows the scheme of the intervention for the EG.

#### The Control Group

Participants assigned to the CG received a booklet in which cognitive games were illustrated. Moreover, suggestions on how to live a healthy life with active aging were provided in the booklet. At the beginning of the trial, the participants in the CG were invited to follow the booklet, play the cognitive games, and follow the suggestions, but they were neither motivated nor contacted during the experiment, unless they wished otherwise.

### The RCT Phases

The PoC procedure was divided into three different phases after recruitment of the participants: baseline evaluation (T0), midterm evaluation (T1; after 3 months from T0), and final evaluation (T2; after 6 months from T0); the aim was to collect data, as described in Textbox 1. All questionnaires and scales at each phase of the PoC were administered through face-to-face interviews by eligible professionals at health care facilities.

**Proof-of-concept (PoC) phases.**

**Recruitment phase:**

The recruitment protocol included general information about the subjects, in particular their health status and cognitive condition. The information was collected with the help of the caregivers/family members, if needed.

**Baseline evaluation:**

This phase consisted of the first real contact with the users and their families before the start of the PoC.

**Second evaluation (after 3 months of use):**

The aim of the midterm evaluation was to collect useful information about the use of the engAGE (Managing Cognitive Decline Through Theatre Therapy, Artificial Intelligence, and Social Robot–Driven) platform after a short period of use for detecting and analyzing technology acceptance and usability issues. Obviously, the control group (CG) was not evaluated at this stage.

**Final evaluation (after 6 months of use):**

The aim of this phase was to collect useful information about the benefits perceived by the users after a meaningful period of use of the engAGE system. The final evaluation was conducted after system deinstallation in order to detect and analyze the impact of the system on the daily life of older adults and their families.

### Recruitment

**Figure 3:**
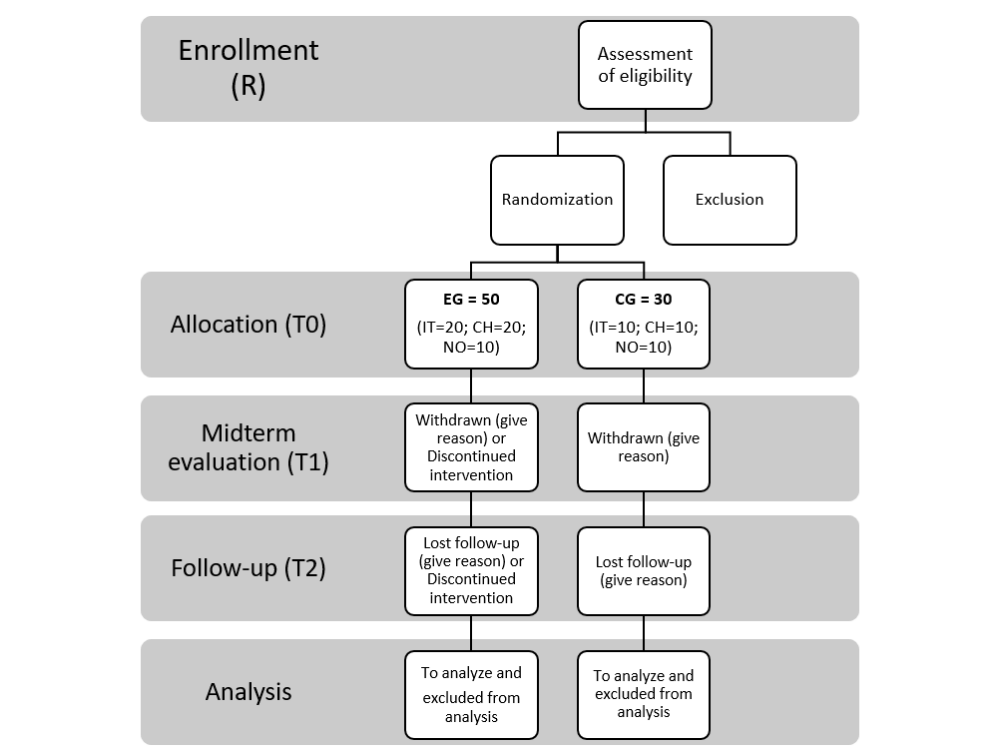
Recruitment methodology flowchart. CG: control group; CH: Switzerland; EG: experimental group; IT: Italy; NO: Norway.

A specific number of people were recruited for the study via health care institutions or health care organizations. Potential participants were evaluated by eligible professionals through face-to-face interviews to verify that eligibility criteria were met. Those who did not meet the criteria or declined participation were excluded. The remaining participants were randomly assigned to the EG or the CG. A randomization technique, conducted by a statistician, based on a single sequence of random assignments was adopted. A list of random numbers was generated by the computer, while subjects were assigned a number based on their order of inclusion in the study. According to this technique, in Italy and Switzerland, the 30 subjects were randomly assigned to one of the two study groups (n=20, 66.7%, in the EG and n=10, 33.3%, in the CG), while in Norway, 20 subjects were equally assigned to the two groups. Next, for both groups, we took careful note of who were lost to follow-up, who discontinued intervention, or who were excluded from analysis. The procedure adopted is summarized in Figure 3.

### Older Adults With MCI

Once informed consent was obtained in duplicate, compliance with the inclusion and exclusion criteria of the study (Textbox 2) were verified, and baseline evaluation was carried out using questionnaires.

**Inclusion and exclusion criteria for people with mild cognitive impairment (MCI).**

**Inclusion criteria:**

- Aged ≥65 years
- Capacity to provide informed consent
- Already diagnosed with MCI
- Global Deterioration Scale (GDS) [24] score<1
- Montreal Cognitive Assessment (MoCA) score [25] between 21 and 25
- Memory Assessment Clinic Questionnaire (MAC-Q) [26] score≥25
- Reisberg scale [27] score between 2 and 4
- Clinical Frailty Scale [28] score between 1 and 3
- Having an informal/formal caregiver to support in carrying out the main daily activities

**Exclusion criteria:**

- Failure to meet inclusion criteria
- Concomitant participation in other studies
- Lack of written informed consent
- Significant visual or hearing impairment

#### Informal Caregivers

Informal caregivers are mostly family members or daily references of patients. Connections between informal caregivers and clients are private and not via a health care organization. Informal caregivers are concerned about the well-being of patients, and they provide emotional or practical support regularly. Although it is easier to live near the patients, informal caregivers do not necessarily need to live close by. Informal caregivers were included in the engAGE project since they play an important role in the daily life of people with MCI (Textbox 3). Moreover, informal caregivers can fulfil caring tasks to reduce the burden of formal caregivers. A good collaboration between informal and formal caregivers is needed as informal caregivers can be the link between client and formal caregiver.

**Inclusion and exclusion criteria for informal caregivers.**

**Inclusion criteria:**

- Being the informal caregiver of an older adult with mild cognitive impairment (MCI)
- Availability of time to participate
- Visiting the patient at least two times a week or living with them

**Exclusion criteria:**

- Failure to meet the inclusion criteria
- Concomitant participation in other studies
- Lack of written informed consent

#### Formal Caregivers

Care professionals or formal caregivers are professionally responsible for dementia care. They are trusted agents for people with MCI and have direct contact with older adults and provide care when needed.

Formal caregivers are case managers, neurologists, psychotherapists, occupational therapists, and nurses. The case manager was responsible for the organization. The neurologist was a specialized medical doctor who diagnosed the disease and prescribed treatment. The psychotherapist led the intervention, whereas the nurses and occupation therapists acted as assistants. These professionals might be especially found in daycare, but they are available in residential home care as well (Textbox 4).

**Inclusion and exclusion criteria for formal caregivers.**

**Inclusion criteria:**

- Over 1-year experience
- Psychologist, neurologist, occupational therapist, or nurse from a health care facility or paid by the participants
- Availability of time to participate

**Exclusion criteria:**

- Failure to meet the inclusion criteria
- Concomitant participation in other studies
- Lack of written informed consent

### Outcomes

The PoC aimed to assess the feasibility of using the engAGE platform as a support tool to counteract cognitive decline in older adults with MCI. For this reason, the primary interest was to assess the impact of the engAGE platform on cognitive capacity. Secondarily, the study also focused on the impact on the social dimension and quality of life, as well as the usability and acceptability of the platform. All the outcomes are presented in Table 1.

**Table 1:** Tools and dimensions of the primary end users’ protocol.

Outcome	Clinical assessment
Perceived stability of cognitive status by older adults	MAC-Q^a^ [26]
Acceptability of the engAGE^b^ platform	UTAUT^c^ [29]
Social connectedness of older adults	UCLA^d^ [30]
Reduction in the caregiver burden of formal and informal carers	Zarit et al [31]
Adherence to intervention	Ad hoc questionnaire
Well-being of informal caregivers	WEMWBS^e^ [32]
Quality of life of older adults	EuroQol 5-Dimension 5-Level (EQ-5D-5L) [33], Quality of Life-Alzheimer’s Disease (QoL-AD) scale [34]
Usability and affordability of the solution of informal caregiver	SUS^f^ [35]
Improving the quality of work of formal carers	Ad hoc questionnaire

^a^MAC-Q: Memory Assessment Clinic Questionnaire.

^b^engAGE: Managing Cognitive Decline Through Theatre Therapy, Artificial Intelligence, and Social Robot–Driven Interventions.

^c^UTAUT: Unified Theory of Acceptance and Use of Technology.

^d^UCLA: University of California, Los Angeles.

^e^WEMWBS: Warwick-Edinburgh Mental Well-Being Scale.

^f^SUS: System Usability Scale.

### Data Analysis

This subsection describes the measurements, statistical tests, and tools with which the data collected in the engAGE PoC were, and are still being, analyzed. The different types of data that were collected in the engAGE project are as follows:

- Credentials: username, password, email address, and similar security information used for authentication and account access in different services of the platform
- Personal data: first name, last name, gender, age, weight, height, email address, contact address, and phone number of older adults in different services of the platform
- Answers to specific questions in self-assessment questionnaires on MEMAS
- Games score from Pepper apps
- Measurements and monitored parameters: weight, heart rate, blood pressure, and physical activities gathered through the monitoring infrastructure
- Historical data of the measurements and monitored parameters for training and using ML algorithms for each older adult in the ML service

In the engAGE testing and evaluation phase, the following data were, and are being, processed:

- Information and results from questionnaires
- Cognitive game scores
- Historical data of measurements collected for each end user
- Any information the end users decided to share through discussions/interviews, as well as data providing feedback from trials
- Outcomes that concern feasibility (ie, the primary interest of the engAGE PoC): recruitment rate (proportion of eligible individuals who consent to participate in the study, ie, those who attended more than 80% of weekly sessions), retention rate (proportion of participants who completed the 6-month intervention period), and adherence to the intervention (average number of interactions with the Pepper robot during weekly sessions, the frequency of use of the MEMAS app, and the activity tracker recording time)

Descriptive statistics (means, SDs, medians, IQRs) will be used to summarize adherence metrics.

Outcomes that concern efficacy of the engAGE intervention were considered exploratory and dealt with the metrics about cognitive capacity, quality of life, social dimension, and stress and care load relief (for caregivers). In particular, to explore the potential efficacy, changes in these domains before and after the intervention were analyzed. Paired *t* tests (for normally distributed data) or Wilcoxon signed-rank tests (for nonnormally distributed data) were used to compare pre- and postintervention scores within each group, while independent samples *t* tests or Mann-Whitney U tests were performed to assess between-group differences in change scores. Moreover, effect sizes (Cohen d or Cliff δ) were calculated to quantify the magnitude of any observed differences. Due to the nature of the PoC, this study was not powered to detect statistical significance. *P* values will be reported without adjustments for multiple comparisons and interpreted with caution. All analyses were planned for descriptive purposes, and only participants with at least 80% of adherence to the intervention were considered for analysis.

The aforementioned analyses were also applied to acceptance and usability measurements (ie, UTAUT and SUS scores) that provided insights into the primary goal of the study: the feasibility of the intervention. Data from the ML service were analyzed to correlate physical activity, sleep parameters, game scores, and self-assessment data. Longitudinal data analysis techniques (eg, mixed effects models) were used to analyze changes in these parameters over time and assess potential correlations with cognitive and quality-of-life outcomes.

The first step in the data analysis dealt with the description of the sample. Continuous variables will be reported as either the mean (SD) or the median (IQR) on the basis of their distribution (assessed using the Kolmogorov-Smirnov test). Categorical variables will be expressed as an absolute number and percentage. Mann-Whitney U tests (for nonnormal distribution) or chi-square tests (for normal or nonnormal distribution) were used to compare independent and dependent variables between the pre- and postintervention conditions, in addition to simple descriptive statistics (means, medians, and SDs, as appropriate).

To verify the achievement of the primary endpoint (ie, MAC-Q score), subscales of the questionnaire were calculated. Means (SDs) or medians (IQRs) of the scores will be reported according to their distribution. Correlation coefficients (Pearson for normally distributed variables, Spearman for nonnormally distributed variables) of the subscales with the other rating scales at each stage of the study and with the main characteristics of the subjects were calculated to check for potential determinants of higher acceptability.

Statistical analyses (ie, quantitative measurements) were performed with RStudio version 4.3.1 (Posit), while qualitative data (ie, interview transcriptions) were analyzed with the Maximum Qualitative Data Analysis (MAXQDA) program.

### Ethical Considerations

#### Ethics Approval

To guarantee the observance of ethics, the principles of the Declaration of Helsinki [36] and Good Clinical Practice guidelines were adhered to, and the study was initiated following receipt of an evaluation and approval by national independent ethics boards in two of the three countries: the Ethic Committee of the IRCCS INRCA in Italy (protocol number 6293/2023) and by the Commission cantonale d’éthique de la recherche (CCER) in Switzerland (project ID 2023-00774). Due to national laws, ethical approval was not needed in Norway, since the study fell within the technological scope. The study protocol is publicly accessible at ClinicalTrials.gov (registration number NCT06302686).

#### Ethical Issues

Technological dependence, especially on artificial intelligence (AI) technologies, represents a major ethical dilemma today for the scientific community. To limit the risk, the European Commission’s international programs have introduced guidelines for conducting studies that require the introduction of a new technology, called *responsible innovation* [37]. The core principles of responsible innovation were also applied in the engAGE project. A strategy underlying the prevention of technological dependence includes different actors around older adults in the process of acquiring skills and daily use of technology. In this way, technological solutions, such as those proposed by engAGE, respond to the definition of a sociotechnological system rather than a technological system, since they are placed in a care context that does not involve the replacement of the caregiver but stimulates the user to play a leading role in the management of their health. The services proposed are intended to support the maintenance or improvement of cognitive abilities through specific activities and do not replace (in whole or in part) the support from professional services. During the installation of the technology, moreover, information is provided to the caregiver about the limits of the technology, which can in no way replace the role of the familiar and formal assistant but only assist in some activities.

During the use of robotic platforms or, in general, technological applications, a general difficulty in distinguishing between the artificial world and reality may occur. Especially in the case of vulnerable populations, such as older adults, the interaction may generate a general feeling of attachment and dependency. To avoid this, engAGE has been designed to not look as a human but to preserve artificial aesthetics, as suggested by the guidelines [38]. In addition, during the first contact with the participants and also during the intervention, researchers are in contact with the users, continuously stimulating awareness about the technological applications, as well as monitoring the appropriateness of the use of the solution in terms of the users’ autonomy, specific needs, and personal preferences. Following the recommendations provided by the Italian Bioethics Board (Comitato nazionale di bioetica, Cnb) of the Italian Ministry of Health [39] on robotics and roboethics, the exit strategy of engAGE aims to do the following:

- Promote adequate experimentation of robotics in field assistance to ensure conditions for physical and psychological integrity of the end user, explaining the risks and benefits, highlighted also in the informed consent form.
- Ensure both equitable access to robotic and general technologies and the use of robots to assist and not to replace humans in order to avoid delegating the irreplaceable human task of care and assistance to the machine.
- Demonstrate that the introduction of robotics in medicine entails always the real consideration of benefits; of the complexity of the change, complete with the structure of the services; and of the economic burden that this entails.

Users who took part in the study did not incur any direct or indirect costs related to the use of the technology platform. The platform was provided to them by the pilot sites and had to be returned to the research team at the end of the trial.

#### Informed Consent for People With MCI

Participants provided written informed consent, even though the acquisition of informed consent from people with MCI is a long-debated issue. Although degenerative neurological syndromes over time lead to a progressive decline in cognitive functions and the ability to express valid consent, the diagnosis of Alzheimer’s disease or MCI does not, in itself, lead to the loss of this ability. The legal capacity and the capacity to act remain, unless proven otherwise, from the age of adulthood until the death of the person. In fact, only a judicial measure can protect an individual with MCI who is unable to provide informed consent by appointing a legal representative. From a neuropsychological point of view, the impairment of executive functions (abstraction ability, problem solving, judgment and criticism, planning, planning, farsightedness, decision-making) is directly proportional to the decrease in capacity both in a general sense and in specific fields: health and economic decisions, driving skills, etc. However, these cognitive functions can be spared in the early stages of the disorder (MoCA score=21-24), unlike other functions that are affected early, such as memory and orientation. It is also common clinical experience that even if unable to understand the contents of a standard informed consent form (which must certainly be simplified), an individual with MCI is often able to express their choices in line with their lifestyle, preferences, and values. This underlines the importance of preserving the possibility for potential participants to use their skills to share possible choices.

Informed consent is a legal condition in which an individual accepts an action that is proposed to them (in our case, active participation in the feasibility study). To be informed, consent must be based on a full understanding of the action itself and the implications it can have. This implies the following:

- Every effort must be made to guarantee and respect any residual capacity for autonomous decisions, considering consent as an instrument through which the subject realizes their autonomy.
- The autonomy of the subject requires that all information be understood.
- The person’s consent presupposes their ability to choose freely based on their preferences, moral values, life stages, and circumstances.

First, it was necessary to inform the older adults with MCI by adapting the information to their cognitive abilities, making every effort so that they could directly or indirectly communicate their preferences. The opinion of family members, for example, could be requested but was considered secondary to that of the patients. Older adults with MCI who gave their informed consent to participate but were not comfortable during the sessions had the option to withdraw from the trial at any time without any consequences.

In the specific case of the engAGE PoC, neither were serious, harmful effects on the patients with MCI foreseeable nor was there bad faith in the treatment proposal. The research teams proceeded to ask the older adults with MCI to provide their informed consent to participate and strive to do the following:

- Ensure that they clearly understood the content of the information sheet and the consequences of their participation.
- Create the best conditions in which they can ask questions and express their will.
- Monitor throughout the course of the trial the persistence of their willingness to participate.

During the clinical interview, the contextual assessment of the ability to express an autonomous choice was carried out, assessing the presence of the following:

- Ability to express a choice
- Ability to understand information relating to consent
- Ability to give due weight to the situation and its possible consequences
- Ability to use information rationally

If this evaluation gave a positive result, informed consent was acquired from the individual. Time and effort were devoted to providing correct and full information, the information sheets and the consent form were read together with the individual with MCI and their caregiver, an opportunity to ask questions was provided, and the best conditions were created to decide. An additional opinion was requested from the main and reference caregivers on whether the individual with MCI should participate in the project and what wishes and feelings about participation might not have been expressed. If the subject showed signs of dissent before and during each training session or showed behaviors that suggested that they were no longer willing to participate, the sessions were terminated, and the consent was automatically withdrawn.

#### Safety and Security Considerations

The hardware devices used are commercial devices and Conformité Européenne (CE) certified. The engAGE platform dashboards for older adults and caregivers are loaded on a tablet held by the users.

Personal data collected during the trial were, and are still being, handled and stored in accordance with the General Data Protection Regulation (GDPR) [40]. All data and documentation related to the trial are stored in accordance with applicable regulatory requirements, and access to data is restricted to authorized trial personnel. The project is committed to the maintenance of participants’ anonymity and confidentiality throughout all procedures, including screening, recruitment, testing, evaluation, and dissemination procedures. Data collection, usage, and storage procedures complied with national laws and the GDPR [40], including the commitment to participants’ right to access information, right to be informed, right to withdraw, and right to data erasure. Moreover, the servers are in the European Union and compliant with the GDPR. Use of the study data is controlled by the principal investigator. Data collection is compliant with the principle of data minimization, that is, the collection of personal information from study participants is limited to what is directly relevant and necessary to accomplish the specific goals of the testing and evaluation work packages. Data entry was carried out using specific software, providing blocks and data entry checks to reduce the number of entry errors. All screening data were discarded upon project completion. During the testing procedures, all visual, auditory, and sensory data that the robot collected and processed to function as planned were discarded after the procedures were completed. The exception to this is the collection of the number of interactions that the robot logged with each participant. However, these interactions were anonymous. All research data will be made openly available for secondary analysis 3 years after project completion. The study findings will be used for publication in peer-reviewed scientific journals and presentations in scientific meetings. Summaries of the results will also be made available to investigators for dissemination within their clinics.

In the case of the engAGE project, a critical area of security is the servers (in the cloud or on the premises) on which the solutions are deployed or data are stored. These have been provided with physical and logical protection. In addition, the communication network architecture implements mechanisms of comprehensive network protection against intrusion, such as an intrusion prevention system, a firewall, and a network antivirus filter. The core system is placed in a secure zone, excluding the necessary communication modules located in the demilitarized zone, enabling data exchange with engAGE services, devices, and components. In the case of communication via an external network, strong mechanisms are required to guarantee the protection of transmitted data, as well as their integrity, confidentiality, and nonrepudiation (eg, Transport Layer Security [TLS]). Authorization procedures have been implemented, specifying who is authorized to access the network and network services (access to services should be possible only for authorized users/devices by providing authentication and authorization mechanisms). Access to individual apps must require a user identifier and authentication (password, authentication certificate). The app functionality available to individual users is limited by each user’s rights. The system architecture includes solutions that eliminate or significantly reduce the system’s vulnerability to attacks, as recommended in the Open Web Application Security Project (OWASP). Data are transferred using REST (Representational State Transfer) application programming interfaces (APIs) using HTTPS. In engAGE, different types of databases are used for storing data. These database servers give the option of encryption at several levels and provide flexibility in protecting data from disclosure (eg, password storage encryption, data partition encryption).

## Results

Recruitment started in October 2023 and continued until April 2024, when there was still time to attend 80% of the trial in the three sites. At the end of recruitment, 40 (81.6%) of the total 49 older adults with MCI were assigned to the EG and 9 (18.4%) to the CG. The PoC started in November 2023 and ended in July 2024. The start and end dates were different across countries because of the various difficulties encountered in organizing the trial and recruiting end users in each site. The efficacy of the engAGE platform will be demonstrated on the basis of the improvement or maintenance of cognitive capacity. In addition, the effects on the quality of life and social connectedness of primary end users, as well as the reduction in the care load and stress of caregivers, will contribute to determining the importance of the platform. Furthermore, the feasibility of the engAGE intervention will be assessed on the basis of the usability and acceptance of the platform, both measured through standardized quantitative scales. Data analysis was completed at the end of 2024, and the results will be published at least by 2026.

## Discussion

### Summary

The engAGE project is an innovative intervention designed to counteract cognitive decline in older adults with MCI by integrating social robots, mobile apps, and wearable sensors into a comprehensive platform. The presented PoC will demonstrate the feasibility of this approach that merges in-facility and at-home technology-based intervention in three European countries (Italy, Switzerland, and Norway). The PoC will also show preliminary efficacy in maintaining cognitive function, which is the primary goal of the engAGE platform. Moreover, a positive impact on the quality of life, physical activity levels, and sleep quality among participants will be demonstrated, as well as acceptability and usability rates of the platform. Regarding secondary end users, the introduction of the engAGE platform is expected to reduce caregivers’ burden and enhance their engagement in the care process.

The engAGE platform addresses critical gaps identified in previous studies on digital health interventions for MCI. Social robots have been explored for their potential to engage older adults with dementia or MCI [17,18]. Although these studies have demonstrated the acceptability of social robots for promoting social interaction and cognitive stimulation, they often focus on single-domain interventions (eg, storytelling or quizzes) rather than a holistic approach combining cognitive training, physical activity monitoring, and caregiver support. In addition, several mobile apps have been developed for cognitive training in older adults with MCI, such as those focused on memory games or problem-solving tasks [13,14]. However, these apps often lack integration with other tools or technologies that address physical activity or social engagement. Similarly, telehealth interventions have shown promise in providing remote cognitive rehabilitation, but they typically do not include real-time interaction [15,16]. In contrast to these existing solutions, engAGE offers a multicomponent intervention that integrates social robotics, mobile apps, and an activity tracker, as well as personalization by ML algorithms. Moreover, the innovative approach of the engAGE platform integrates in-facility services and home-based intervention. This comprehensive approach aligns with recommendations from recent systematic reviews emphasizing the need for multidomain interventions to address the complex needs of older adults with MCI [41,42].

### Limitations

Although the engAGE project aims to represent an important step toward developing integrated digital health solutions for older adults with MCI, several limitations should be acknowledged. Despite the study being structured as an RCT, the sample size was not computed, and the number of end users enrolled was relatively small (N=80), limiting the generalizability of findings to larger populations. Due to the focus on the feasibility and initial efficacy of the intervention, the findings should be used to inform future sample size calculations and refine intervention protocols for future studies that aim to demonstrate the efficacy of the engAGE platform. Participants were recruited from health care organizations and daycare centers located in city areas, introducing a potential bias: people with less access to health and digital services were not considered. However, to make results more generalizable, the study was conducted across three European countries (Italy, Norway, and Switzerland) that show differences in cultural and health care contexts.

The duration of the study (ie, 6 months) was minimal, as it was unlikely to observe significant changes in cognitive capacity or the caregiver burden. Moreover, ML algorithms are designed to personalize interventions based on user data, and the study duration could not be sufficient to fully profile individual preferences or needs, making the personalization inefficient. Finally, the complexity of the platform due to the integration of several technologies, services, and ML algorithms introduced ethical concerns and exposed the users to risks in terms of data protection. However, both data protection and ethical issues were considered and addressed within the engAGE project to guarantee users’ security and safety. Thus, the presented PoC should be followed by a larger structured RCT that involves a larger sample to appreciate the significant impact of the engAGE platform, including personalization functionalities, both on end users and on national health care systems.

## Appendix

Multimedia Appendix 1 Proposal review and acceptance document from the Active Assisted Living Programme, funded by the European Union.

## Abbreviations

AAL: Ambient and Assisted Living
CG: control group
EG: experimental group
engage: Managing Cognitive Decline Through Theatre Therapy, Artificial Intelligence, and Social Robot–Driven Interventions
GDPR: General Data Protection Regulation
ICT: information and communication technologies
IRCCS INRCA: Istituto di Ricovero e Cura a Carattere Scientifico – Istituto Nazionale di Riposo e Cura per Anziani
MAC-Q: Memory Assessment Clinic Questionnaire
MCI: mild cognitive impairment
ML: machine learning
MoCA: Montreal Cognitive Assessment
PoC: proof of concept
RCT: randomized controlled trial
SUS: System Usability Scale
UCLA: University of California, Los Angeles
UTAUT: Unified Theory of Acceptance and Use of Technology
WEMWBS: Warwick-Edinburgh Mental Well-Being Scale

## Data Availability

The datasets generated during this study are available from the corresponding author upon reasonable request. Popular scientific dissemination, conference proceedings, related research papers, and public deliverables including the findings of the project are public and accessible at the official engAGE (Managing Cognitive Decline Through Theatre Therapy, Artificial Intelligence, and Social Robot–Driven Interventions) website [43].
