# Supplementary material for: Managing Cognitive Decline Through a Social Robot–Based Intervention: Protocol for the engAGE Proof of Concept and Randomized Controlled Trial
Source: JMIR Res Protoc. 2025 Sep 2;14:e67601. doi: 10.2196/67601 (PMC12441641; doi:10.2196/67601)
Supplement: Multimedia Appendix 1 [file resprot_v14i1e67601_app1.pdf]

The Active Assisted Living Programme

Evaluation Summary Report (ESR) -

Evaluation-call-2020

PROPOSAL: Managing cognitivE decliNe throuGh theatre therapy, Artificial intelliGence and social robots drivEn interventions

aal-2021-8-159-CP

1. RELEVANCE AND SCOPE — 3 / PASSED

The proposal addresses the Call challenge well, namely to provide an ICT based solution developed to support the healthy and active living and the general wellbeing of individuals affected by cognitive decline. The proposal is aligned with the AAL areas, particularly the Health&Care Area (Health and Care Prevention), Vitality and Abilities (Cognitive Abilities), Information and Communication (Consulting Coaching and Assistance). The proposal outlines clearly the main targeted purchasers/consumers of the ICT solution, which addresses the Institutionalized market.

The example scenario is very convincing and presented with several details about different end users: the description of the implementation of the solution is realistic, and it addresses very well all different situations occurring in the daily life of an individual affected by cognitive decline. The scenario is very helpful in clarifying the needs of the different end users, and relations among them.

The primary end-users are older adults with Mild Cognitive Impairment (MCI) living independently and supported by caregivers, male or female over 65 years needing care assistance to self-manage and support their cognitive function. The secondary end-users are formal & informal caregivers and healthcare organizations.

The proposal clearly identifies how the project explores new ideas, concepts, and approaches for ICT-based solutions for older adults. This “product” is an integration of different components that are in the market nowadays, but the innovative point, and therefore the Unique Selling Point (USP), is the complete approach to the self-management of older adults’ cognitive decline by joining non-invasive IoT and self-reporting monitoring, machine learning-based assessment, and social robot-driven interventions in terms of coaching, cognitive function stimulation, and social interaction facilitation.

Regarding the uniqueness of the proposal, the project's rationale is clear as to objectives and foreseen activities. The Consortium demonstrates a good understanding of the challenges that are well presented in three main aspects: low engagement of older adults with traditional cognitive stimulation application, the coaching support and social interaction, and the traditional screening methods. These are very relevant and meaningful goals, but it is not sufficiently clear how this project will achieve this.

The proposal addresses well the lack of context information and the need of a holistic approach when dealing with people with cognitive decline.

The proposal does not fully identify the comparative advantage of the proposed solution over existing solutions. This is because the proposers do not specify similar systems for senior assistance, including a comparison between them; based on the target group, the characteristics and the current price (this point is important for the economic Key Performance Indicators of the project and the future sustainability).

The proposal sets out well how the team plans to investigate the wishes and needs of end-users concerning the proposed solution. This assessment is based on the involvement of end-users, experts and stakeholders during all the project through focus groups, meetings and trials. The proposal sets out well the approach to end-user engagement, including (i) a clear plan for how it will build strong collaborations with end-user organizations. (ii) a clear plan for how it will use novel and improved approaches for involving end-users. (iii) a clear plan for how it will validate benefits for end-user organizations, enhancing the processes used by those organizations. (iv) a good case that the project concept has strong market potential. These assessments are based on the involvement of end-users in such levels as engAGE services co-design and development and the field trials evaluation of the engAGE prototype (~ 80 primary end-users). Also, caregivers (~ 55 secondary end-users) and stakeholders (2 seniors daycare centres) are included in the methodology, and the instruments and variables to be measured are well described.

Regarding the key performance indicators, these are sufficiently outlined, with clarity and accuracy, both as to quantity and quality aspects: overall, they meet the expected goals and results. The description divided per project year is very helpful. Among the key indicators, the one called "Launching the customers Behavior" presents inconsistency and one critical point: while in year 1 and 2 the target is based on users express intention to “purchase” the final solution and the final product, in year 3 the target is 50% of users express intention to “purchase/own” the system. Moreover, to “purchase” and to “own” are two different intentions based on different elements (for ex. the costs impact) for the assessment: the measurability of the results remains a crucial issue in this case, also considering the robot solutions that always face difficulties in reaching a wide distribution in the market. Finally, beyond

users' acceptance, other qualitative indicators could have been included. A shortcoming is that there are no KPIs included that measure directly the most importance goals of the project, with a focus on engagement, better social interaction, and accurate methods for measuring and detecting changes in cognitive abilities.

2. IMPLEMENTATION - QUALITY OF PROPOSED SOLUTION AND WORKPLAN — 4 / PASSED

EngAGE focuses on two groups of users: seniors with MCI, formal and informal caregivers and the end-users' perspectives are considered in sufficient detail, focusing on satisfaction and self-esteem and not only illness and limitations. The proposal defines adequately which types and numbers of end-users will be included and how they will be recruited. A considerable number of end-users (~240) will be involved in the proposal, in all chains of value, with an extensive level of activities including analysis, design, test and validation. The consortium will follow the 'Design for All' and 'Universal Design' principles during the design, implementation, evaluation, and business modelling. This involvement approach is well justified in the proposal. The methodology for the research and piloting consists of iterative co-design. The project sets out a convincing case that it will create a realistic environment to test the prototype. This assessment is based on the provided testing plan. This end-user involvement approach is well-targeted to the project objectives and the example scenario. However, a special attention should be given to test the Human-Robot Interaction with older people.

A shortcoming is that there are no pilots defined that will directly measure the most relevant goals of the project, ie. Achieving better engagement, better social interaction, and accurate measurement of cognitive decline based on machine learning techniques.

Critical stakeholders are involved in an adequate level of activities on the value and commercialization of the prototype's tested and validated prototype. The project activities are well targeted to meet the proposed goals. There is a coherent description of the aims and tasks and between the technological development and the testing. Concerning the commercialization activities, the SME participants are responsible for 50% of the total effort. The other partners are research entities, end-users centered and universities. Key partners are involved in the commercialization to push the product into the market two years after the project. The partners responsible for commercialization are sufficiently engaged in all key activities leading up to market launch.

Overall, the proposal shows consistency of actions, with a very effective matching of end-users' involvement and the general objectives and expected results and the end-user involvement description is consistent with the initial example scenarios. Participation is adequately outlined with effective methods in all the different parts of the projects: design and development, business modelling, implementation of the pilot, testing and evaluation. Inclusion and exclusion criteria are very well described. A good level of details is provided for the testing and evaluation activities.

Recruitment of end users is always a challenge, because of the time-consuming terms of recruitment. The Consortium demonstrates to be aware of this issue, and they will address it with a reduced size of the control group. This point could impact the investigation and it should be clarified how this would be managed in terms of comparability of results, or, it would be needed to clarify better a profile of "quasi-experimental" design. Again, this is even more crucial, when the experiment will involve people with cognitive decline and when the test will include robot solutions.

Another major shortcoming is that the proposed workplan does not consider sufficiently what parts of the envisioned solution could be considered medical devices. In particular, the software that will track and measure cognitive decline has a reasonable chance of being considered a medical device under de new MDR regulation. This would have a large impact not only on the workplan, but also on the commercialization and business plan.

The proposal is appropriately structured into five strongly interwoven work packages, with tasks coherent with the project's objectives (i) Technology development and integration; (ii) End-user continuous involvement and co-creation; (iii) Testing and Evaluation; (iv) Dissemination & Exploitation strategy; (v) Project Management. The deliverables and milestones are well related to and coherent with the project objectives. Regarding the resources, they are well balanced and proportionate with the scale and complexity of the work described. The proposal mentions the algorithms for Monitoring and Big Data, ML-based Cognitive Decline Assessment Service, Social Robot Coaching and Cognitive Stimulation, Communication Platform, and Intelligent Personalization. The expected TRL should at the end of project development be TRL 7-8.

The proposal does not sufficiently explain the necessity or specific advantage of the new engAGE platform for the algorithms to be implemented, nor does it specify the requirements posed by the algorithms to the platform. The framework is largely built on existing technology and devices already developed: an ADL monitoring infrastructure will build upon TelluCloud; the cognitive decline assessment will benefit from big data and a machine learning platform developed by TUC; the communication platform will be based on MEMAS software developed by KARDE, while the robot that will be programmed for interventions is Pepper or Temi. This is adequate for an AAL project. However, the proposal does not sufficiently cover the state of the art well in the most critical areas to carry out the proposed work. Also, it does not sufficiently present state of the art related to AAL systems. Moreover, the proposal is general when identifying the gap in the market. For example, identifying some potential competitors and existing technologies provides limited information about the alternative process and potential outcomes to the existing ones.

The business development as well is very well explained; the technology, standards and interoperability are well addressed, with detailed description, more information should be provided, again, in the testing part, where more information on how and where the robot will be part of the testing for all enrolled users is needed. Potential commercial partners are also well described, and their participation is well described in all three phases - design, deployment and marketing. Business partners have adequate resources, with a +50% of "Person Month". All critical stakeholders are involved, and their participation is adequate for the expected results and final outcome of the proposal.

Regarding one of the main component of the solution, that is the social robot coaching and cognitive stimulation, more information should have been provided on similar solutions, both in terms of acceptance and in terms of wide market potential, considering also what has been already developed and tested in past projects. Moreover, some more detail on the part related to theatre and drama storytelling would have helped in understanding better the potential of the proposal (as these aspects are

included also in the title of the proposal). The proposal presents limited information about barriers and facilitators of the potential scaling up.

3. IMPLEMENTATION - QUALITY OF CONSORTIUM AND PROJECT MANAGEMENT | 4 / PASSED

The collaboration and management structures (decisions and conflict resolution systems) are clearly set out. The way in which the competence of the project coordinator and partners is mapped to the tasks to be performed is set out clearly, and the way in which the capabilities and innovative potential of the consortium will be mobilized appears convincing. There is a Steering Committee, a project coordinator, a Technical Manager, an Impact Manager, a National (Local) Ethics Manager, an Advisory Board, a legal, Ethical and Security Committee and WP leaders. The complementarity between the consortium members is fully justified because of the specific tasks of each technological partners and the description of who is going to carry out the pilots with end-users' partners.

The resources required (personnel, facilities, networks, etc.) to undertake project activities and deliver the expected outcomes will be fully available to the consortium once they receive funding. The consortium is built up from a mixture of 6 partners whose endeavor will be distributed between scientific research, innovation, and end-user involvement. It includes 1 university (TUC), 2 end-users organizations (HUG, INRCA), 3 industrial partners (IRIS, TLU, KRD). The Consortium has expertise in artificial intelligence, decision making, big data analytics, ontologies and semantic Web, knowledge engineering, deep neural networks, mobile applications development, natural language processing and data analytics.

The approach builds on available knowledge and tools developed through previous projects. Their complementary role is crucial in exploiting the scientific potential and translating it into innovations. Also, the SMEs partners identified good experience in the B2B and B2C market, with considerable impact in different countries and stakeholders. The allocation of tasks to the partners is in line with their specific expertise and the proposal objectives. The proposal includes a coherent Gantt diagram. The funding requested from AAL, including a breakdown of costs and major budget requests, is well set out and is in line with the expected results. The person months for each partner is coherent with the tasks associated. This assessment is based on the comparison of the budget for the trials and the technological development. The description of how resources will be deployed to the various work activities and through the project timescale is well set out and generally convincing. The total cost of 1 921 930,0 € is balanced to the estimated 222 PM project effort. Also, the requested funding of 1 269 090 € for 30 months is in line with the expected results and impact. A shortcoming is that resources assigned to research tasks are more difficult to assess. For example, it is very difficult to judge whether resource estimates to develop the machine learning models are adequate or not. The duration of the testing phase is adequate in principle, in line with the expected duration for this type of study. However, considering the target population and key components of the solution, such as the robot, the partners must be aware that more time may be required to get realistic feedback. Compliance is always an issue, and in particular when it concerns users with cognitive decline and interaction with a robot. Finally, the difference in the tests of the various components in terms of time should also be further clarified, such as the part of drama storytelling and interaction.

A good list of risks is included in the project application. These risks span all relevant areas including end-user, market uptake, project management and technological risks. Overall, however, the likelihood/impact of risks seem to be rated as rather low. The consortium may underestimate some of the challenges of the project, especially those challenges that have to do with increasing user engagement and reliably being able to measure cognitive decline. Another shortcoming is that risks related to medical device regulation are not mentioned. It seems likely though that some components of the solution would be considered as medical device under MDR.

Intellectual property rights (IPR) are addressed well, covering the main relevant aspects. The consortium Agreement generally regards the main aspects – exploitation, intellectual property rights, confidentiality, ownership of knowledge and access rights and the pre-existing know-how on use and dissemination; however, an appropriate conflict resolution mechanism is lacking.

The consortium addressed all relevant ethical issues and demonstrates high awareness of the relevant national and European rules and regulations. This is because they include informed consent in all the trials and consider normative and data protection (GDPR). It also demonstrates a good understanding of technical and other relevant rules and regulations and how they vary across the European regions the consortium will be operating in and intend to deploy their proposed ICT solution.

4. IMPACT - POTENTIAL IMPACT OF PROPOSED SOLUTION ON QUALITY OF LIFE | 4 / PASSED

The proposal describes clearly the expected impact of the solution. This includes the quality of life of end-user groups, social and ethical aspects, methodological aspects and business development aspects.

The impact is very well explained through a QoL framework that includes different dimension: Physical functioning, performance of discretionary activities, bodily wellbeing, social interaction. This gives a strengthened view of the intervention logic and its impact. The description could be strengthened with available information on the impact of robot solution already tested and the long-term acceptance and compliance of primary end users. The key for the social impact is the increase of the autonomy of the end-users and their relations with caregivers. Also, cost savings is another economic benefit for society.

Additionally, the impact can be expected in people who need special assistance, such as people with inadequate health literacy or mild cognitive impairment, young adults that may suffer from MCI. The proposed solution represents a significant improvement in the quality of life of the targeted end-user group (primary and secondary end-users). This is because the Quality of Life measure will be done during the trials with end-users and caregivers through focus groups, tests, and questionnaires. EngAGE is expected to have the potential to improve the quality of life and well-being of seniors and their formal and informal caregivers. It will improve their communication, independence and integration in society. The project can be useful not only for older people but also for formal and informal careers. EngAGE will reduce workload and care-related stress.

A convincing case is made that the proposed solution, if successfully developed, can indeed have major impact on quality of life of primary and secondary end-users. However, the consortium seems to be overly optimistic about the possibility to reach all these goals.
The exit strategy should be improved with some additional information on the alternative solution that might be offered to users or with additional information on cost and financing for people that want to keep using the proposed solution. The drop-out management description should also be improved with additional information.
The impact on the service model is well explained, with a direct approach and comparing the usual care and the innovation proposed. Anyway, it looks more focused on components (that have been already presented in the previous parts of the proposal), and less on organizational elements. The cost benefit analysis presents very clear indications of cost dimensions and qualitative benefits, but it looks weaker in quantifying the quantitative dimensions of benefits. The application makes a case that expensive clinical tests to measure cognitive decline could be replaced by the machine learning techniques to be developed in this project. If indeed these machine learning techniques can be developed and proven to be working well, then indeed this would be a major benefit. However, it seems very challenging to get this done, and such techniques may very well be considered medical devices.
The proposal identifies barriers to achieving the project goal and its steps to address barriers; they are also included in the risk management plan. However, the proposal does not identify sufficiently how it plans to adapt the solution to different social, organizational, and funding systems across Europe. Moreover, it does not describe the expected impact of the solution on savings for the social care system sufficiently. The proposal sets out clearly the framework considerations (such as regulation, standards, public acceptance, and value chain cooperation) that may determine the project's impact.

5. IMPACT- POTENTIAL IMPACT OF PROPOSED SOLUTION ON MARKET DEVELOPMENT	3 / PASSED

The composition of the consortium is adequate to have skills and experience that will permit an effective market entry, while the greatest challenge appears to be the direct selling to primary end users. It is clear that direct potential customers are the people with cognitive decline and their families, but the proposal states that the main target groups will be healthcare professionals and commercial stakeholders, that will indirectly affect the adoption of the solution by older adults and family members / informal caregivers.
The proposal describes clearly the expected benefits of the solution. This includes increased autonomy, dignity, and self-confidence for a longer time, added value for the secondary clients, reduced workload, care safety, and social inclusion.
The strength of the innovation is the product delivered on both the regulated market (B2B2C main path) and private market (B2C secondary).
The detailed activities towards market introduction are no known yet, but the consortium already has a good view on them. TELU will lead the commercialization activities supported by IRIS and KRD, TELU being experienced in the development and sales of digital healthcare services, social robots, and IoT applications. End-users INRCA and HUG all act as expertise centers for care providers and cognitive decline management, hence they are eager to promote the care services for older adults with MCI within their networks.
A first iteration of the business model canvas is available that summarizes the business model.
The solution that will be offered is sufficiently clear, and there is a comparison available with competing solutions which demonstrates differentiation.
Some indications are given in the proposal related to possible sales prices (subscriptions) both for the institutionalized market as well as for the consumer market. There is a rather weak description of why the targeted users will want to use the product. This is because the older people with MCI may not be interested in the solution. The initial costs are presented. To deploy the engAGE system and to buy the robot - 18000 euro for Pepper or 6000 euro for Temi. For older adults and family caregivers, the option of leasing the robot and using the engAGE system (200 euro/month for Pepper or 80 euro/month for Temi) is foreseen. The risk of low willingness to pay should be described in more details and monitored carefully.
Also, there is no view at this moment on the cost structure of the business and therefore profit-loss predictions are not available yet. This is a shortcoming as it seems a very large risk to invest in this project if it is not at least reasonably sure that the envisioned value proposition translates into sufficient willingness to pay and therefore a feasible revenue/cost structure. Especially in a situation where some components could be considered medical devices, it is important to understand whether reasonable revenue forecast will be sufficient to carry the costs of certification and market introduction.
The time to market (maximum 2 years) and the proposed timeline is credible. However, there is not enough information to conclude, with evidence, that EngAGE will be a clear cost-effective AAL solution. External stakeholders required for market success are set out poorly. The impact on strengthening the competitiveness and growth of companies was not clearly explained.
The proposal sets out well the description of IPR handling and any other legal issues that may be applicable. It is noted that the proposal describes the future “rules” for the future GA and also takes into account the background and foreground knowledge required for the exploitation of the product. The instruments to achieve the target groups are sufficiently identified as well as the criteria to evaluate the impact of dissemination.

6. REMARKS	NOT RATED

TOTAL SCORE: 18
